# Measurement of Circulating Tumor Cells to Track Hepatocellular Carcinoma Progression After Liver Transplantation-Case Report

**DOI:** 10.3389/fonc.2021.760765

**Published:** 2021-10-20

**Authors:** Yuan Deng, Lei Sun, Heng Liang, Bojing Cui, Caimei Cui, Dan Zhao, Arabella Wan, Guohui Wan

**Affiliations:** ^1^ National-Local Joint Engineering Laboratory of Druggability and New Drug Evaluation, National Engineering Research Center for New Drug and Druggability (Cultivation), Guangdong Province Key Laboratory of New Drug Design and Evaluation, School of Pharmaceutical Sciences, Sun Yat-Sen University, Guangzhou, China; ^2^ LABVIV Technology (Shenzhen) Co., Ltd, Shenzhen, China; ^3^ Zhongshan School of Medicine, Sun Yat-Sen University, Guangzhou, China; ^4^ Department of Gastrointestinal Surgery, The First Affiliated Hospital, Sun Yat-Sen University, Guangzhou, China

**Keywords:** circulating tumor cells, hepatocellular carcinoma, tubular adenoma, metastasis, liver transplantation

## Abstract

Management of patients with hepatocellular carcinoma (HCC) largely relies on surgery and other systemic therapies. However, the poor diagnosis of cancer recurrence or metastasis can lead to a high frequency of treatment failure. Thus, factors that can predict disease status and prognosis of patients need to be identified. Circulating tumor cells (CTCs) are known to accurately predict survival of patients. Here, we report a case in which CTCs successfully predicted the progression of metastatic colon polyps after interventional therapy for HCC. A 48-year-old man was diagnosed with HCC with moderate differentiation in 2016 and subsequently underwent orthotopic liver transplantation. Discharge medications were continued with immunosuppressants (tacrolimus) and antiviral drugs (Titin). In 2018, a colon polyp, a type of tubular adenoma, was detected and surgically removed. However, in 2020, the same tubular adenoma recurred. During cancer progression, CTC counts were measured to monitor the status of metastasis, and a positive correlation was noted between the dynamic change in CTC counts and cancer response (metastasis or recurrence). When diagnosing the metastatic adenoma, the number of cytokeratin-positive CTCs was significantly increased; however, it dropped to zero after the polyp was surgically removed. The same change in CTC counts was observed during the second recurrence of the adenoma, and a subgroup of CTCs, cell surface vimentin-positive CTCs, was significantly increased. The CTC count dropped to an undetectable level after the surgery for the first time. In summary, we presented a clinical case in which CTC counts could predict disease progression during HCC metastasis. Thus, CTC counts should be measured after liver transplantation in patients with HCC for diagnosis and clinical decision-making as it is effective in monitoring cancer progression.

## Introduction

Hepatocellular carcinoma (HCC) is a major type of primary liver cancer and has high morbidity and mortality worldwide. In China, HCC was the second leading cause of cancer-related mortality in 2020 (1). Early stage HCC has no obvious symptoms and can be rarely detected; however, generally, most patients are accurately diagnosed in the immediate or advanced stage. Compared with the increasing number of patients with HCC, limited progress has been made in identifying an effective treatment for HCC (2).

Currently, the practice pattern of liver cancer has changed from a single local treatment to multidisciplinary comprehensive treatments such as surgery, ablation, interventional therapy, targeted therapy, immunotherapy, and other organic combinations (3). Although surgical resection remains the most preferred treatment for HCC, more than 90% of patients with HCC in China have a background of hepatitis B infection, combined with cirrhosis, liver dysfunction, multicentric occurrence of tumors, early spread, and early metastasis. Furthermore, only approximately 20%–30% of patients can undergo surgical resection. For patients who cannot undergo radical surgeries such as liver transplantation, tumor resection, or percutaneous radiofrequency ablation, transarterial chemoembolization or transarterial radioembolization is the core treatment. Although patients with HCC are generally managed with surgery, critical problems such as relapse or spread can occur in patients within several months.

In clinical studies, intrahepatic metastasis is the most common route of metastasis in liver cancer. However, autopsy revealed that extrahepatic carcinoma metastasis, the other assignable one, had a metastasis rate of approximately 40%–71.6%. There are several pathways of metastasis in liver cancer, including blood channels, lymphatic channels, direct dissemination, and local diffusion.

Circulating tumor cells (CTCs), a subset of cells in the blood of patients with solid tumors, are responsible for tumor metastasis (4). The cancer cells from the primary site metastasize *via* the bloodstream in the form of single CTCs or multicellular groupings, CTC clusters (CTM). Moreover, emerging evidence has suggested that CTCs, including cytokeratin-positive CTCs (CK^+^ CTCs) and cell surface vimentin-positive CTCs (CSV^+^ CTCs) are detected as supplementary data in liquid biopsy. CK^+^ CTC refers to tumor stem cells or CTCs undergoing epithelial-mesenchymal transformation (EMT), which most commonly metastasize. While intracellular vimentin expresses in normal mesenchymal cells that limits its role as a CTC marker, Satelli et al. identified the cell surface vimentin that was only associated with cancer cells, making it as a potential EMT CTC marker (5). Furthermore, the molecular and biological features of CTCs help with prognostic prediction and therapeutic selection during clinical decision-making (6); thus, CTCs play a crucial role in clinical practice.

Compared with traditional imaging methods, disease status and prognosis of patients can be indicated by CTC counts in the early stages of metastasis, and using liquid biopsy, overall survival of patients can be accurately predicted. Thus, in this study, we report a case in which CTCs successfully predicted the progression of metastatic colon polyps after interventional therapy for HCC.

## Case Description

In December 2016, a 48-year-old man, with a history of positive for hepatitis B surface antigen (HBsAg), hepatitis B e antibody (HBeAb), and hepatitis B core antibody (anti-HBC), started an irregular “Bolidine” antiviral therapy 7 years ago. He has no family history or psychosocial history. The examination found that AFP increased significantly in the short term from 40ug/L in July 2016 to 241.37ug/L in August 2016, and the upper abdominal MR showed multiple nodular hepatocellular carcinoma with multiple intrahepatic metastases. Subsequently, the patient presented with HCC was successfully treated with an orthotopic liver transplantation, following which he was prepared for discharge with the long-term use of immunosuppressants (tacrolimus) and antiviral drugs (entecavir maleate). Positron emission tomography and computed tomography revealed an irregular mixed density with uneven deposition of lipiodin in the anterior and lower parts of the right liver lobe (near the gallbladder fossa), together with slightly increased marginal metabolism. The liver showed no obvious abnormality, however, the residual active tumor may still exist in the liver.

**Figure 1:**
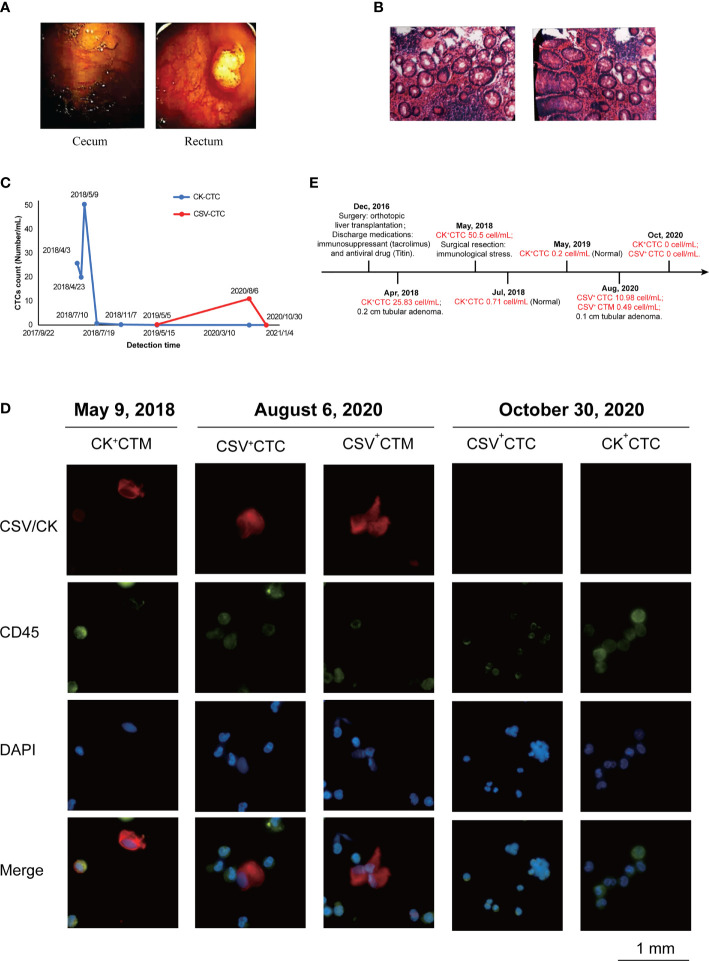
Monitoring CTC count to track HCC progression after liver transplantation. **(A)** Electronic colonoscopy of the cecum and rectum in April 2018. Images revealed a spherical subpedicle polyp of about 0.6cm x 0.8cm in size in the cecum, with a smooth surface; and 8cm away from the anus, there are 2 polyps in the rectum, with the larger size of 2.0cm x 1.6cm. **(B)** Pathological biopsy observed under a microscope in August 2020. Images revealed under the light microscope, part of the epithelium showed mild to moderate atypical hyperplasia, with increased cell levels, but no clear malignant changes were seen. **(C)** Changes in circulating tumor cell (CTC) counts (number/mL) after metastasis. The summarizes results of CTC analysis in the present case from April 2018 to October 2020. The specimen showed the counts (number/mL) of CK+ CTCs changed from April 2018 to November 2018 obviously and then the counts (number/mL) of CSV+ CTCs also showed variations from May 2019 to October 2020. **(D)** Representative immunofluorescent images of CTCs obtained separately after blood analysis of the present case on May 9, 2018, August 6, 2020, and October 30, 2020, using Cellab Thomas I system (LABVIV, Shenzhen). CTCs were defined as captured cells that were cytokeratin positive (CK+) or vimentin +, CD45-, and DAPI+. The cytokeratin positive (CK+) or vimentin + were stained as red color; CD45+ was stained as green color and DAPI+ was stained as blue color. **(E)** Treatment timeline of patient from December 2016 to October 2020.

In April 2018, electronic colonoscopy revealed no abnormalities in the mucosa of the terminal ileum, opening of the appendix, and ileocecal valve. However, a spherical polyp measuring 0.6×0.8 cm in size was observed in the cecum, and the surface was smooth (**Figure 1A**). The intestinal lumen of the ascending colon, transverse colon, descending colon, and sigmoid colon was normal, and the plica appeared regular. The mucosal surface was smooth and moist, and the vascular network was clear. No abnormal secretions, erosions, ulcers, or masses were observed. However, two polyps were noted approximately 8 cm from the messenger of the rectum. The larger polyp measured 2.0 ×1.6 cm in size, and its surface was rough and erosive (**Figure 1A**), which was confirmed by the pathological images (**Figure S1**).

Results of the laboratory panel and tumor marker levels assessed during diagnosis in April 2018 are provided in **Tables 1**, **2**. Hepatic metabolism examination revealed that the number of indirect bilirubin increased, while the total protein levels decreased. No abnormality associated with existing hepatitis B was noted. Alfa-fetoprotein (AFP), carcinoembryonic antigen (CEA), and free prostate-specific antigen (FPSA) levels were normal. However, cancer antigen 19-9 (CA19-9) values showed an abnormal increase. CA19-9 is a tumor marker associated with pancreatic cancer, gallbladder cancer, colorectal cancer, and gastric cancers and is also known as gastrointestinal-associated antigens. Because CA19-9 has high sensitivity and specificity for most cancers, its positive rate is between 85% and 95% during diagnosis, which decreases with postoperative improvement. All examinations revealed the appearance of a 0.2-cm-sized tubular adenoma, which is an effective precancerous marker of colorectal cancer. After surgically resecting the adenoma, reduplicative examinations were conducted twice, and the results showed that the CA19-9 level increased to 71.88 U/mL in June 2018 and decreased to 68.89 U/mL in August 2018. The last examination in August 2018 revealed a normal CA19-9 level.

**Table 1:** Biochemical laboratory values in April 2018.

Parameter	Value
ALT	15 U/L
AST	23 U/L
γ-GGT	40 U/L
ALP	79 U/L
AST/ALT	1.53
Total blirubin	17.8 μmo/L
Direct blirubin	6.3 μmol/L
Indirect blirubin	11.5 μmol/L
DB/IB	0.55
Total protein	63.7 g/L
ALB	42.8 g/L
Globulin	20.9 g/L
A/G	2.05
a-1 AFU	21 U/L
Cholinesterase	9251 U/L
Leucine arylamidase	34 U/L
Adenosine deaminase	7 U/L
Total bile acid	3.76 μmol/L

ALT, Alanine transaminase; AST, Aspartate transaminase; γ-GGT, Gamma-glutamyltransferase; ALP, Alkaline phosphatase; AST, Aspartate transaminase; ALT,alanine transaminase; DB, Direct bilirubin; IB, Indirect bilirubin; ALB, Albumin; A/G, Albumin/Globulin; α-L AFU, α-L-Fucosidase.

**Table 2:** Laboratory values of immunity (Tumor and HBV) in April 2018.

Parameter	Value
AFP (tumor)	4.050 IU/ml
CEA	2.92 ng/ml
EB-Nal-IgA (tumor)	–
CA 19-9 (tumor)	66.52 U/ml
CYFRA21-1 (tumor)	3.17 ng/ml
NSE (tumor)	11.8 ng/ml
CA72-4 (tumor)	1.08 U/ml
PSA (tumor)	1.24 ng/ml
FPSA (tumor)	0.329 ng/ml
HBsAg (HBV)	0.471 COI
HBsAb (HBV)	227.7 IU/ml
HBeAg (HBV)	0.104 COI
HBeAb (HBV)	0.85 COI
HBCAB (HBV)	0.011 COI

AFP, alpha fetoprotein; CEA, carcino-embryonic antigen; EB,Epstein-Barr; NaI, Nuclear antigen; CA 19-9, Carbohydrate antigen19-9; CYFRA21-1, Cytokeratin 19 fragment; NSE, Neuron specific enolase; CA72-4, Carbohydrate antigen 72-4; PSA, Prostate specific antigen; FPSA, Free prostate specific antigen; HBsAg, Hepatitis B surface antigen; HBsAb, Hepatitis B surface Antibody; HBeAg, Hepatitis B e Antigen; HBeAb, Hepatitis B e Antibody; HBcAb, Hepatitis B core Antibody.

In August 2020, pathological biopsy was performed to confirm the diagnosis of tubular adenoma with low-grade intraepithelial neoplasia. Observation by naked eyes revealed one grain grayish tissue measuring 0.1 cm in diameter. Under light microscopy, some epithelia showed mild to moderate atypical hyperplasia with increased cellular levels, but no definite malignant changes were observed (**Figure 1B**).

Moreover, changes in CTC counts (number/mL) provided evidence that the appearance and increased number were associated with recurrence or metastasis during monitoring (**Figure 1C**). In April 2018, the patient was diagnosed with colon polyps, with 25.83 cells/mL of CK^+^ CTCs. In addition, because of the immune stress caused by surgery, the number of CK^+^ CTCs unconventionally increased to 50.5 cells/mL. Continuous monitoring in July revealed normal CTC counts. Moreover, the patient completed a periodic examination in May 2019, and no abnormalities were noted. However, emerging evidence has showed that the amount of CK degraded during the interstitial (EMT) phase in the CTC formation process, which may result in false negative signal in the detection of CTC. Compared with the epithelial tumor marker (CK), cell surface vimentin (CSV) was found with higher enrichment in CTCs with a more efficiency in the detection. Consistently, the same increasing tendency of CSV^+^ CTCs was detected in August 2020. Under microscopic examination, the correlation between CTC counts and metastasis was clearly demonstrated. Finally, 2 months after surgery for adenocarcinoma, no evidence for CSV^+^ CTCs or CK^+^ CTCs was found. Thus, the clinician hypothesized that CTCs originated from the adenocarcinoma locus. The common trends noted in the total counts of CSV^+^ CTCs and CK^+^ CTCs are presented in **Table 3**.

**Table 3:** The summarizes results of immunofluorescent CTC in the present case from April 2018 to October 2020.

DATE	Blood sample	DAPI^+^ cells	CD45^-^ cells	CK^+^ CTC	CK^+^/DAPI^+^	CSV^+^ CTC	CSV^+^/DAPI^+^
	Volume (ml)	Total Number	Total Number	Total Number	Percentage(%)	Total Number	Percentage (%)
4/3/2018	6	1465	1264	155	10.58	–	–
4/23/2018	6	1730	1655	70	4.05	–	–
5/9/2018	6	3480	3157	303	8.71	–	–
7/10/2018	5.6	4230	4128	4	0.09	–	–
11/7/2018	5.2	4011	4101	1	0.02	0	0
5/5/2019	5	755	760	0	0	1	0.13
8/6/2020	6	17599	17824	0	0	67,2 (3,3)	0.38
10/30/2020	5.5	2700	2822	0	0	0	0

Immunofluorescence was performed to validate the number and morphology of CTCs isolated from the patient’s blood sample. The isolated cells were differentially identified using an epithelial marker (CK) or cytoskeleton marker vimentin, a hematopoietic white blood cell marker (CD45), and the existence of a cell nucleus (4,6-diamidino-2-phenylindole, DAPI), as previously described (7). The cells were subsequently subjected to image analysis using a fluorescence microscope. CTCs were found to be DAPI positive, CD45 negative, vimentin positive, or DAPI positive and CK positive, with different nuclear morphologies from leukocytes. (**Figure 1D**). The results were quantitatively reported as the number of CTCs per 5 mL of whole blood. The immunofluorescent images showed CTCs before surgery on August 6, 2020. Two months later, the immunofluorescent images demonstrated the disappearance of CTC images. The timeline of the patient treatment showcased the complete date and progression from the episode of care (**Figure 1E**).

## Materials and Methods

### Boold Samples and CTC Enumeration

5mL of blood were collected by K2 EDTA vactutainer (IMPROVE MEDICAL, China), and centrifuged at 500×g for 10 minutes. The plasma was discarded and the equal volume of cell preservation solution (LABVIV, China) was filled. CTCs suspension were enumerated by Cell Sort Kit (LABVIV, China) according the manufacturer’s instructions.

### Cell Staining

Cytokeratin stain: Cells were fixed with 4% paraformaldehyde for 10 minutes, permeabilized with 0.1% PBS-Triton X-100 for 10 minutes. Cells were incubated with rabbit polyclonal anti-pan CK antibody(ab217916, Abcam, USA) and mouse monoclonal anti-CD45 antibody(ab8216, Abcam, USA) for 1 hour at room temperature. Subsequently, cell were incubated with Goat polyclonal Secondary Antibody to Mouse IgG H&L (Alexa Fluor^®^ 488) (ab150113, Abcam, USA) preadsorbed at 1/1000 dilution, Goat polyclonal Secondary Antibody to Rabbit IgG - H&L (Alexa Fluor^®^ 594) (ab150080, Abcam, USA) at 1/1000 dilution and Nuclear DNA was labelled with DAPI for 1 hour. Image was acquired with Fluorescence inverted microscope (Nikon Ti2, Japan) (8).

Vimentin Stain: Cells were fixed with 4% paraformaldehyde for 10 minutes. Cells were incubated with rabbit polyclonal anti-vimentin antibody (ab137321, Abcam, USA) and mouse monoclonal anti-CD45 antibody (ab8216, Abcam, USA) for 1 hour at room temperature. Subsequently, cells were incubated with Goat polyclonal Secondary Antibody to Mouse IgG H&L (Alexa Fluor^®^ 488) (ab150113, Abcam, USA) preadsorbed at 1/500 dilution, Goat polyclonal Secondary Antibody to Rabbit IgG - H&L (Alexa Fluor^®^ 594) (ab150080, Abcam, USA) at 1/500 dilution and Nuclear DNA was labelled with DAPI for 1 hour. Image was acquired with Fluorescence inverted microscope (Nikon Ti2, Japan) (9).

### HE Staining

Samples from the patient were placed into the pre-prepared fixative (10% formalin, Bouin’s fixative). After deparaffinized with xylene, the fixed samples were hydrated through decreasing concentration of alcohol baths (100%, 90%, 80%, 70%) and distilled water. The slides were stained in hematoxylin for 3-5 minutes and subsequently dipped into 1% acid alcohol (1% HCl in 70% alcohol) for 10 seconds followed by washing. They were counterstained in 1% Eosin Y for 10 minutes and subsequently dehydrated in the increasing concentration of alcohols (10).

## Discussion

HCC management is more complex because of its asymptomatic nature in the early stage, poor prognosis, and easy recurrence or metastasis (11). Orthotopic liver transplantation is one of the most common HCC therapies. According to statistical data, liver transplantation has a high 5-year survival rate of over 75%, and the recurrence rate is less than 10%. However, because of poor prognosis owing to tumor size, degree of vascular invasion, extrahepatic lymph node metastasis, and other factors, treatment failure has become a critical problem (12). An increasing number of patients have recurrence and metastasis, which is generally confirmed by tissue biopsy or other invasive surgeries.

In this scenario, simpler and more convenient detection modalities are needed in clinical practice. Determining CTC counts in peripheral blood, known as “real-time liquid biopsy”, in patients with solid tumors has recently been performed because it can be performed frequently, easily, and less invasively compared with other detection methods such as tissue biopsy. In addition to diagnosis, CTCs play an important role in clinical decision-making and monitoring of disease progression. However, under normal circumstances, CTCs are rarely found in the blood, at levels typically >1 billion cells. In addition, unlike normal tumor cells, they are more fragile. Therefore, the enrichment of CTCs with high purity and recovery is a great challenge (13–15). Over the past few decades, epithelial cell adhesion molecule (EpCAM), a universal tumor marker, have been widely used in CTC detection (16–19). To date, it seems clear that CTCs are highly heterogeneous and dynamically change their shape incessantly during blood or lymph circulation. More importantly, carcinoma cells that pass through a partial or complete EMT process are no longer detectable by EpCAMs in peripheral blood (20). Other markers, such as CTCs, also originate from epithelial cells, including CK or human epidermal growth factor receptor 2 (5). As a large amount of CK in CTC degrades during the EMT phase, another novel marker, CSV, is replaced with CK in some cases (21, 22). Therefore, CK^+^ CTCs and CSV^+^ CTCs are the two parameters for CTC counts (23–25).

Interestingly, we observed that certain clinical conditions could influence CTC counts, where changes in CTC counts reflect tumor response to therapy. Our patient had HCC and was successfully managed by liver transplantation. When tubular adenoma was diagnosed, high CTC counts were noted during liquid biopsy in April 2018. Later, higher CTC counts were also detected, which could have been caused by immune stress after surgery. Statistically, most patients who have previously undergone polypectomy have high CTC counts. However, in our patient, CA19-9 and CK^+^ CTC values returned to the normal range within months, following which the patient returned to a healthy condition.

Laboratory data and colonoscopy examinations also implicated the absence of abnormalities in existing hepatitis B or other infections. AFP, CEA, and FPSA levels were within the normal range, except for CA19-9 levels, which were found to be abnormally elevated. The conventional biomarker showed a potential indication for metastasis, while CTC counts and tissue biopsy accurately confirmed the diagnosis. However, tubular adenoma recurrence occurred in 2020. Based on CTC detection, it was obvious that the tumor responded negatively to previous therapy, with increasing CSV^+^ CTC values. Subsequently, the tubular adenoma was verified using a microscope. Moreover, the number of CTCs consistently disappeared after resection for the second time. The patient was thus followed up based on CTC counts to predict cancer progression.

Adenomas can be detected in a timely manner *via* CTC detection. In our patient, after 2 months, the CTC counts returned to the normal range, which lasted for 2 years. This shows a relationship between the number of CTCs and disease progression. For instance, CTC sequencing revealed that the number of CTCs was correlated with the single-nuleotide polymorphism (SNP) of intestinal adenocarcinoma by CTC sequencing (26). Adenomas usually appear as benign neoplasms. However, the current survey found that the risk of colorectal cancer greatly improved if relatives have a medical history of adenomas (27). Moreover, after liver metastasis, a higher risk of bowel cancer occurs with the continued use of immunosuppressive drugs. Research conducted by Harvard Medical School also found that tumor metastasis could be effectively blocked through CTC clearance in the peripheral blood (28), which also proved the importance of CTC in subsequent treatments. Emerging evidence has supported that CTC can act as an adaptive prognostic marker; however, limitations remain in the field. First, we need to select the most suitable molecular biomarker for CTC detection to decrease false-negative results. Second, we need to establish specific standards for CTC detection in prognosis to serve better in clinical practice.

In conclusion, we report a case of HCC treated with liver transplantation in which CTCs were detected during the entire process of metastasis cancer therapy. After liver transplantation, the patient was diagnosed with multiple adenomatous polyps of the colon in 2018, during which the number of CK^+^ CTCs was detected to have a 3-fold increase. In 2020, CSV^+^ CTM counts also increased rapidly, and the polypus of colon was surgically removed after the indication. The elevated CTC counts indicated the potential metastatic status of HCC and was evidenced by the detection of polyps in the colon. The detection of CTCs could be used as a biomarker in cancer research, filling the gap of unanimity across clinical studies for accurate prediction during metastasis progression.

## Patient’s Perspective

During the whole treatment timeline, I had gone through a very tough and even miserable experience. Too many tests applied on me in order to assist diagnoses and therapies, but I have been optimistic towards all the routine tests requested by the doctor. Except from them, I have kept monitoring the CTCs in my body for several times from 2018 to 2020. Although no abnormalities were found in the lungs and liver from routine tests, it alerted me that I should undergo additional gastrointestinal examination after my CTC number increased in time, which had prevented further deterioration. Since I had successful treatment with the identified colon polyp, I was allowed to leave the hospital and made it home. Every time, I was grateful for the CTC detection and the subsequent treatment. I could not imagine how things would go if I did not perform these monitoring in April 2018, and I would not be able to talk about my situation and feeling now.

## Data Availability Statement

The original contributions presented in the study are included in the article/**Supplementary Material**. Further inquiries can be directed to the corresponding authors.

## Ethics Statement

Written informed consent was obtained from the patient for publication of this case report and accompanying images.

## Author Contributions

GW and CC generated conception and designed this study. YD, LS, HL, BC, DZ, and AW developed the methodology and performed the assays. YD and AW analyzed and interpreted the data. BC, CC, and DZ provided administrative, technical, or material support. YD, AW, and GW organized the data and wrote the manuscript. The study supervisors were GW and AW. All authors contributed to the article and approved the submitted version.

## Funding

This work was supported in part by grants from the Guangzhou Science and Technology Planning Program (202002020051), Guangdong Basic and Applied Basic Research Foundation (2019A050510019, 2021B1515020004).

## Conflict of Interest

Authors BC, CC, and DZ were employed by the company LABVIV Technology (Shenzhen) Co., Ltd.

The remaining authors declare that the research was conducted in the absence of any commercial or financial relationships that could be construed as a potential conflict of interest.

## Publisher’s Note

All claims expressed in this article are solely those of the authors and do not necessarily represent those of their affiliated organizations, or those of the publisher, the editors and the reviewers. Any product that may be evaluated in this article, or claim that may be made by its manufacturer, is not guaranteed or endorsed by the publisher.

## Supplementary Material

Supplementary Figure 1 The pathological images of the polyp in April 2018 which was related to **Figure 1**.
