# Supplementary figures and images for: Measurement of Circulating Tumor Cells to Track Hepatocellular Carcinoma Progression After Liver Transplantation-Case Report
Source: Front Oncol. 2021 Oct 20;11:760765. doi: 10.3389/fonc.2021.760765 (PMC8565924; doi:10.3389/fonc.2021.760765)

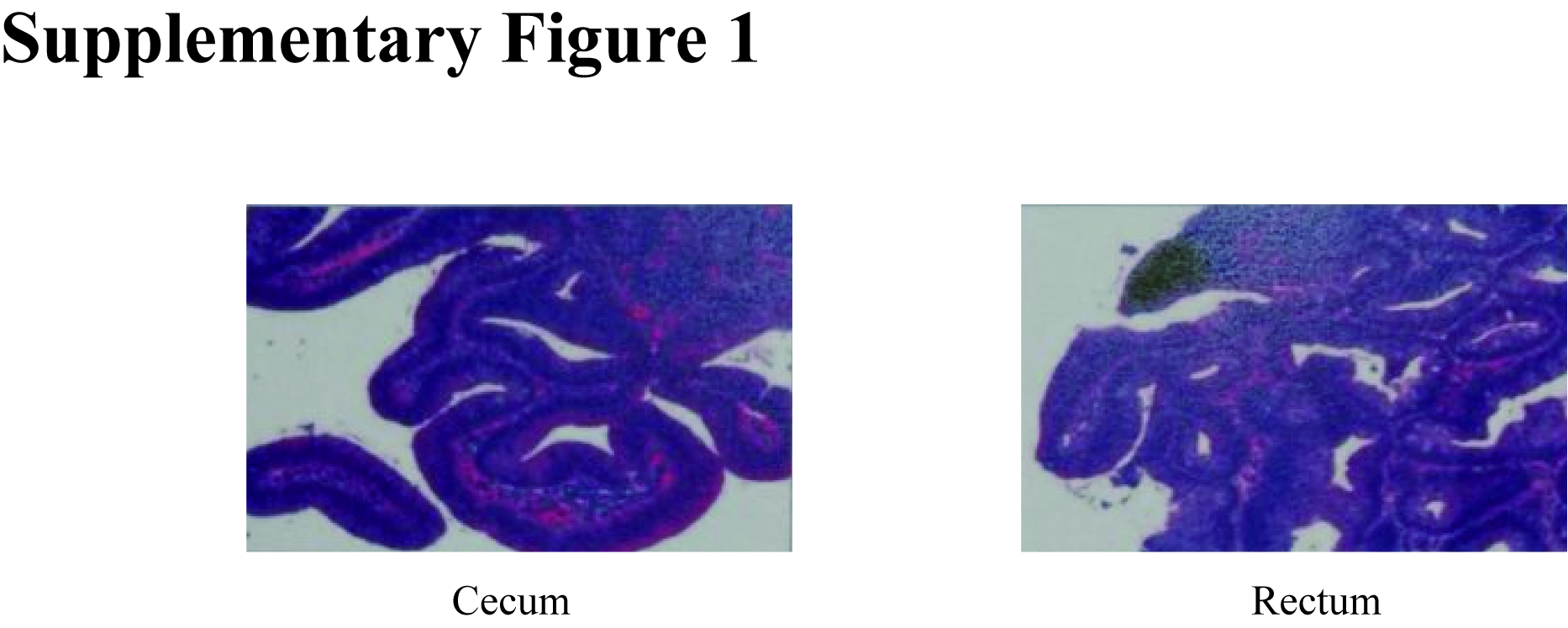

Supplement: Supplementary Figure 1 — The pathological images of the polyp in April 2018 which was related to Figure 1 . [file Image_1.tif]
